# Supplementary material for: Impact of patterns of language use and socio-economic status on a constructed response Situational Judgment Test (SJT)
Source: PLoS One. 2023 Aug 1;18(8):e0289420. doi: 10.1371/journal.pone.0289420 (PMC10393151; doi:10.1371/journal.pone.0289420)
Supplement: Attachment — Submitted filename: Response to Reviewers.docx [file pone.0289420.s001.docx]

# PONE-D-23-08475 - Language Use and SES - Response to Reviewers

July 10, 2023

| **#** | **Comment** | **Response** | **Line reference** |
| --- | --- | --- | --- |
| **Journal Requirements** | | | |
| 1 | Please ensure that your manuscript meets PLOS ONE's style requirements, including those for file naming. The PLOS ONE style templates can be found at  <https://journals.plos.org/plosone/s/file?id=wjVg/PLOSOne_formatting_sample_main_body.pdf> and  <https://journals.plos.org/plosone/s/file?id=ba62/PLOSOne_formatting_sample_title_authors_affiliations.pdf> | The style requirements have been reviewed and the manuscript matches those provided in the templates to the best of our knowledge. Specifically, the headings style has been updated and the citation and reference format has been modified throughout. | NA |
| 2 | Thank you for stating the following in the Competing Interests section:  “SL, LC, MA and DR have no disclosures to declare. XP, VH, and AM disclose  while writing this article they were salaried employees of Acuity Insights which administers a situational judgment test, Casper. The authors receive no reimbursements, fees, or funding related to this study or its outcomes.”  Please confirm that this does not alter your adherence to all PLOS ONE policies on sharing data and materials, by including the following statement: "This does not alter our adherence to PLOS ONE policies on sharing data and materials.” (as detailed online in our guide for authors <http://journals.plos.org/plosone/s/competing-interests>). If there are restrictions on sharing of data and/or materials, please state these. Please note that we cannot proceed with consideration of your article until this information has been declared.  Please include your updated Competing Interests statement in your cover letter; we will change the online submission form on your behalf. | We have added the requested statement to the Competing Interests section:  *“This does not alter our adherence to PLOS ONE policies on sharing data and materials.”* | NA |
| 3 | In your Data Availability statement, you have not specified where the minimal data set underlying the results described in your manuscript can be found. PLOS defines a study's minimal data set as the underlying data used to reach the conclusions drawn in the manuscript and any additional data required to replicate the reported study findings in their entirety. All PLOS journals require that the minimal data set be made fully available. For more information about our data policy, please see <http://journals.plos.org/plosone/s/data-availability>.  Upon re-submitting your revised manuscript, please upload your study’s minimal underlying data set as either Supporting Information files or to a stable, public repository and include the relevant URLs, DOIs, or accession numbers within your revised cover letter. For a list of acceptable repositories, please see <http://journals.plos.org/plosone/s/data-availability#loc-recommended-repositories>. Any potentially identifying patient information must be fully anonymized.  Important: If there are ethical or legal restrictions to sharing your data publicly, please explain these restrictions in detail. Please see our guidelines for more information on what we consider unacceptable restrictions to publicly sharing data: <http://journals.plos.org/plosone/s/data-availability#loc-unacceptable-data-access-restrictions>. Note that it is not acceptable for the authors to be the sole named individuals responsible for ensuring data access.  We will update your Data Availability statement to reflect the information you provide in your cover letter.  –  The [PLOS Data policy](http://www.plosone.org/static/policies.action#sharing) requires authors to make all data underlying the findings described in their manuscript fully available without restriction, with rare exception (please refer to the Data Availability Statement in the manuscript PDF file). The data should be provided as part of the manuscript or its supporting information, or deposited to a public repository. For example, in addition to summary statistics, the data points behind means, medians and variance measures should be available. If there are restrictions on publicly sharing data—e.g. participant privacy or use of data from a third party—those must be specified. | We have revised the Data Availability Statement as there are ethical and legal restrictions pertaining to the release of these potentially personally identifiable data.  In summary we are unable to share these data for the following reasons:  **1 - Third party data** - Data from this study are from educational records belonging to the learners (as per FERPA legislation) and cannot be distributed by the researchers as per ethical review. As such, the following have been included in the Data Availability Statement.  A description of the data set and the third-party source   - The study used data from two medical schools (Rutgers Robert Wood Johnson Medical School and Wake Forest School of Medicine). - Data includes: Applicant identity characteristics (race, socioeconomic status indicators) and educational attainment information (exam and assessment scores)   Verification of permission to use the data set   - The study was approved by Wake Forest School of Medicine IRB (IRB00073284) and Rutgers Institutional Review Board (Pro2022001657). The ethical review boards determined that the study meets the criteria for a waiver of HIPAA authorization according to 45 CFR 164.512, accordingly informed written or verbal consent was not obtained.   All necessary contact information others would need to apply to gain access to the data   - Wake Forest School of Medicine IRB   - [irb@wfu.edu](mailto:irb@wfu.edu)   - IRB00073284 - Rutgers Institutional Review Board   - [IRBOffice@research.rutgers.edu](mailto:IRBOffice@research.rutgers.edu)   - Pro2022001657   **2 - Human research data -** The data set includes potentially sensitive information that could be used to re-identify an individual based on demographic characteristics and examination dates and scores. As such, ethical restrictions have been set to limit data sharing only to individuals involved in the study. The Data Availability Statement has been amended accordingly.  Explain the restrictions in detail   - data contain potentially identifying or sensitive learner information (demographics and test dates and scores)   Provide contact information for a data access committee, ethics committee, or other institutional body to which data requests may be sent   - Wake Forest School of Medicine IRB   - [irb@wfu.edu](mailto:irb@wfu.edu)   - IRB00073284 - Rutgers Institutional Review Board   - [IRBOffice@research.rutgers.edu](mailto:IRBOffice@research.rutgers.edu)   - Pro2022001657 | NA |
| 4 | Please include your full ethics statement in the ‘Methods’ section of your manuscript file. In your statement, please include the full name of the IRB or ethics committee who approved or waived your study, as well as whether or not you obtained informed written or verbal consent. If consent was waived for your study, please include this information in your statement as well. | The following was added the methods section:  *The study was approved by Wake Forest School of Medicine IRB (IRB00073284) and Rutgers Institutional Review Board (Pro2022001657). The ethical review boards determined that the study meets the criteria for a waiver of HIPAA authorization according to 45 CFR 164.512, and therefore informed written or verbal consent was not obtained.* | p.5, l.17 |
| **Reviewer 1** | | | |
| 1 | Thank you for the opportunity to review your manuscript. It was well written, included a logical flow, and was very useful for those who utilize SJTs. I have no comments because I truly did not find any concerns with the information presented.  The only consideration may be details about the statistical model for greater clarity for those who are not as familiar with the statistics, but it is not necessary. | Thank you very much for your review and kind words. |  |
| **Reviewer 2** | | | |
| 1 | The research contains a large sample size that has the power to yield meaningful results, however, differences in size of groups should be recognised. | Thank you for your review.  Indeed, the overall sample size was sufficient as you note.  Regarding differences in sample size, variance across groups was consistent and supported the application of the model as identified by non-significant Bartlett’s test, and inspection of boxplots and Q-Q plot distributions.  In addition to identifying the sample size for each group in Table 2, we have added the following to provide context about the potential implications with respect to unequal representation of the population.  *While sample size and variance across groups support the application of the model, it is possible that the balanced representation of the U.S. population in this study incompletely reflects the relationship between Casper performance and sociodemographic factors. Unfortunately, these distributions are reflective of the medical school applicant community. For instance, 20% of applicants were identified as disadvantaged by AAMC EO status and 2% of applicants indicated having a parent with less than high school degree. Therefore, these findings are more likely to be directly applicable fields where similar demographic profiles are observed in the applicant pool.* | p.14, l.1 |
| 2 | Authors acknowledge the limitation in their sample being US only. It would be beneficial to acknowledge the implications of this on the generalizability of the results to different cultures and contexts that use Casper. | The following has been added to the limitations section:  *These findings may also differ by geography. For instance, in Canada and Australia where Casper is also used there are different patterns in the distribution of race, degree of socioeconomic disadvantage, and patterns of language use. For instance,* [*Leduc and colleagues*](https://journalhosting.ucalgary.ca/index.php/cmej/article/view/72402/54829) *(2021) found similar trends in Casper performance across many demographic groups, however French language use and proficiency was an area of significant investigation in the Canadian context. As such, the origin of these individuals and how that interacts with the region in which they apply are important areas of future investigation.* | p.13, l.16 |
| 3 | The inclusion criteria for SES is quite rigorous (based on five different indicators (gross family income, parental highest education, fee assistance program recipient, family receiving financial aid during childhood, Pell grant recipient)) and the categories used for both patterns of language use (PLU) are logical. However, they argue that candidates with ESL (English second language) are more likely to be immigrants which is associated with lower SES which is a questionable argument. I would also argue that the research referenced (28, 29) does not clearly justify the claim. | Thank you for bringing this to our attention:  We have modified the argument and updated the references as follows:  *Moreover, data from the* [*2019 U.S. Census Bureau*](https://www.census.gov/content/dam/Census/library/publications/2022/acs/acs-50.pdf) *indicates households with limited English-speaking ability disproportionately represent lower income families. While 17% of households not in poverty had limited English-speaking compared to 36% of households in poverty.* | p.4, l.11 |
| 4 | The control variables (race, gender, school applied) are appropriate to the experiment. | Thank you |  |
| 5 | Overall, I think the data generally does support the conclusions that bilingualism was associated with better Casper performance compared to English monolinguals and ESL students. However, there is a lack of explanation for the finding that high SES was associated with better Casper performance than low SES. I would encourage the authors to suggest why this might be the case? The discussion needs to be strengthened in its commentary about the findings in general and possible underlying caudal factors, alongside suggestions for future research. | Thank you for highlighting this shortfall. We have added the following to the discussion on why SES was associated with better Casper performance and directions for future work.  *Consistent with previous work of other assessments, the current study found an association between SES and Casper scores (Reardon, 2013). And like Lievens and colleagues (2016), these differences were smaller in the SJT than what is typically observed with academic evaluations. This gap is thought to exist for several reasons. First, individuals with lower SES generally have had less access to opportunities due to financial and social resource constraints affecting educational and experiential opportunities including mentorship, volunteering, and extra-curriculars to name a few (Crosnoe and Muller, 2014; Griffin and Hu, 2015). Compounding this opportunity gap are the strained mental and emotional experiences many who live with lower SES have (Jury et al 2017). These psychological barriers include being more likely to feel greater emotional distress, challenging feelings of belonging and imposter syndrome in college, and less perceived support from family. All of which may contribute to the trend in lower educational attainment (Jury et al 2017). Lastly, the structure common in U.S. higher education favor people from higher SES backgrounds. The practices, behaviors, and cultural ideals of higher SES households are centered in higher education, leaving people from families with lower SES othered and in unfamiliar territory. (Becker et al., 2017, Calarco, 2014). While there is no real basis for any SES group difference in capacity or aptitude of situational judgment, the gaps in opportunity, elevated psychological barriers, and centering of higher-SES values have real effects and requires improvement. Future work should investigate a wide range of SES proxies and how their associated experiences have impacted applicants (Festin et al., 2017). These learnings can be used to inform the selection process, modify educational values, and improve evaluation measures.* | p.10, l.8 |
| 6 | The use of a hierarchical linear regression makes sense to see how much of the variance in Casper scores can be explained by the different predictors. The way in which variables were imputed into the hierarchical model is logical (demographic confounding variables first, followed by the predictor variables in separate steps followed by the interactions), allowing for an understanding of the amount of variance explained in Casper scores by each of the predictors separately while controlling for confounding variables. | Thank you. |  |
| 7 | • I also believe there is an error in the note in table 5. It says that the variance explained by the demographic variables is 46%. This is not suggested within the table. | Thank you for highlighting this typographic error. We have adjusted it to read 4.7% as it is reflected in the table. | Table 5 |
| 8 | • I am a little confused by the numbers that have been reported. Why is explanatory power reported? R2 and R2 change is not appropriately considered. The explanatory power reported looked like a multinomial logistic regression? Could they be confusing the 2? Is it just a post hoc calculation? This needs clarification. | Thank you for this question. To clarify, the R-square reported in Table 5 is a goodness-of-fit measure for each of the regression models included in the hierarchical regression analysis. It indicates the percentage of the variance in the Casper scores that can be explained by the predictors in each model. By reporting the R-square for each model in the hierarchical regression analysis, we can assess if adding PLU and DSD improves the model’s ability to explain the variance in Casper score.  The model comparison results reported: “*Model comparisons showed that adding PLU significantly improved the model’s explanatory power (χ²(2) = 69.86, p < .001)*” and “*Model comparisons showed that adding DSD significantly improved the model’s explanatory power (χ²(1) = 61.22, p < .001)*” are testing if adding PLU and DSD significantly improved the R-squares.  R-squares reported in Table 5 are calculated in the standard way in linear regressions (1-(sum squared regression/total sum of squares)). Logistic regression does not have an equivalent to the R-square in linear regressions, and pseudo R-square is usually reported and used to facilitate interpretation.  Hopefully this elaboration can clarify the reviewer’s question. | NA |
| 9 | • The language is simple, easy to follow and appropriate. Overall, the article flows well and I consider it well written. It is a bit difficult to follow when the authors go from referring to SES to DSD, it would be beneficial for this section to be made clearer. | We have clarified the use of SES and DSD throughout the manuscript and revised the section that explains the relationship between SES and DSD to read as follows:  *First, to implement a more sensitive and reliable measure of SES (Fenton et al., 2016), a composite measure of SES was calculated based on work by Jerant and colleagues (Henderson et al., 2020; Jerant et al., 2015). This was done to allow SES to be presented as a continuous indicator – Degree of Socioeconomic Disadvantage (DSD). To build the DSD indicator, the following five variables were used to predict applicants’ AAMC SES status (education and occupation (EO) indicator for an applicant’s parent(s): EO1/2 vs EO3/4/5) in a logistic regression: 1) gross family income, 2) highest parental education, 3) Federal Pell Grant recipient, 4) AAMC Fee Assistance Program (FAP) recipient, 5) family receives financial aid during childhood. Predicted probabilities of being identified as EO1/2 (the SES disadvantage status in the AAMC application) were calculated based on the regression model. To evaluate the fit of the DSD indicator, the distinguishing ability of the model was evaluated using the Area under the Receiver Operator Characteristic (ROC) Curve (AUC). With acceptable distinguishing ability, DSD was used in the next step of the analysis to address the primary question.* | p.7, l.6 |
